# Clinical Characteristics of Gastric Duplication in Children

**DOI:** 10.3389/fped.2022.857056

**Published:** 2022-03-28

**Authors:** Fei Liu, Xiaogang Xu, Menglong Lan, Boyuan Tao, Zijian Liang, Jixiao Zeng

**Affiliations:** Department of Pediatric Surgery, Guangzhou Institute of Pediatrics, Guangzhou Women and Children’s Medical Center, Guangzhou Medical University, Guangzhou, China

**Keywords:** gastric duplication, children, foregut, laparoscopic, congenital

## Abstract

**Background:**

To investigate the clinical characteristics of gastric duplication (GD) in children.

**Methods:**

The clinical data of 17 children with GD who were treated in our hospital from July 2015 to June 2021 were analyzed retrospectively. There were 8 males and 9 females, aged from 2 months to 11 years. All children underwent laparoscopic GD resections and postoperative pathological diagnosis was GD. In addition, we searched and analyzed the literature on GD in children from 1 January 2011 to 31 December 2021 from the PubMed, EMBASE, and Cochrane Library databases.

**Results:**

Gastric duplication was more common in females, with the most common cystic type occurring in the greater curvature of the stomach. Vomiting is the most common clinical manifestation. Ultrasound is an effective method for the early screening of GD. In this study, one patient who had multiple GDs underwent laparoscopic cystectomy and mucosectomy, one patient was converted to open surgery, and all other children underwent laparoscopic cystectomies. The time to oral intake was 2.3 ± 1.0 days (range: 1–4 days), and the postoperative hospital stay was 5.7 ± 1.7 days (range: 2–9 days). All children were followed up for 6–77 months and had an uneventful recovery with the resolution of the preoperative symptoms.

**Conclusion:**

Gastric duplication in children lacks specific clinical manifestations, and the preoperative diagnosis rate is not high, so surgical exploration combined with pathological examination is often needed to make a clear diagnosis. Laparoscopic cystectomy can achieve good therapeutic results.

## Introduction

Gastric duplication (GD) is a rare congenital malformation of the gastrointestinal tract, with an incidence rate of 17/1,000,000 (1). Clinically, GD patients may have abdominal pain, vomiting, an abdominal mass, weight loss, and other symptoms, but the lack of specific clinical manifestations, imaging manifestations and laboratory tests make the diagnosis difficult (2, 3). Therefore, it is easy to miss the diagnosis or have a misdiagnosis. At present, the diagnosis of GD still requires surgical exploration combined with pathological examination. Considering that GD may be accompanied by serious complications such as bleeding, perforation, obstruction, and malignant transformation, most studies suggest that once a GD is diagnosed, it should be removed as soon as possible (4–6). This study retrospectively analyzed the clinical data of 17 cases of GD in our hospital from July 2015 to June 2021 and reviewed relevant literature to explore the characteristics of clinical manifestations, diagnosis and treatment of GD in children to reduce the preoperative misdiagnosis rate and to improve the level of diagnosis and treatment of this disease.

## Materials and Methods

The clinical data of 17 children with GD confirmed by surgery and pathology from July 2015 to June 2021 were collected. There were 8 males and 9 females. Their ages ranged from 2 months to 11 years, and their median age was 24 months. All children firstly underwent abdominal ultrasound examination, then computed tomography (CT) and/or angiography were added to assist in the diagnosis of some children whose ultrasound diagnosis was unclear and to assist in treatment plan development. Eight cases (47.06%, 8/17) were diagnosed before operation, including seven cases diagnosed prenatally and one case postnatally. Eight cases (8/17) were diagnosed by ultrasound, seven cases (7/16) by CT, and three cases (3/6) by angiography. The pathological diagnostic criteria for GD included: (1) the cystic wall is adjacent to the gastric wall; (2) the smooth muscle in the cystic wall is connected with the smooth muscle of the stomach; and (3) the cystic wall is lined with gastric epithelium or other types of intestinal mucosa (7, 8). Duplications not located on the stomach were excluded from this study. The present study got the approval from the Institutional Review Board of Guangzhou Women and Children’s Medical Center (approval no. IP-2018-008). Medical histories of the patients were collected during their first routine visit. Informed written consent was acquired from their parents or guardians and the research was conducted in compliance with the World Medical Association Declaration of Helsinki.

### Operative Techniques

The preoperative preparation: abdominal ultrasound, CT, and/or upper gastrointestinal angiography were performed to investigate the size, location, and adjacent relationship of the cyst. The patients were fasted routinely and had indwelling nasogastric tubes and urinary catheters after anesthesia.

Surgical procedure: the umbilicus was cut, and a 5 mm trocar was placed using direct vision. The method of establishing a pneumoperitoneum was the same as that in our previous study (9). Under laparoscopic monitoring, the second and third trocars were placed in the left and right middle abdomen, and 2-0 sutures were used to suspend the falciform ligamentum to better expose the operative field. If necessary, 3-0 Prolene sutures were used to suspend the gastric wall, or an operating forceps was added to the left upper abdomen. All patients underwent laparoscopic exploration to determine the cyst position, freedom and relationship with the surrounding tissues.

**FIGURE 1:**
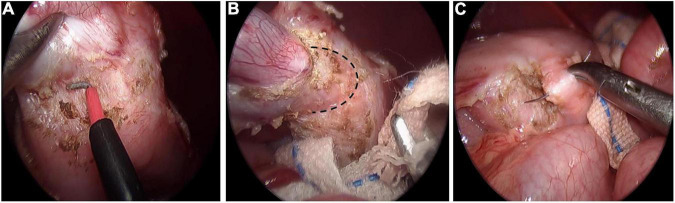
Laparoscopic resection of gastric duplication. **(A)** Separation of GD by using electric hook. **(B)** Blunt dissection to locate the boundary (black dotted line) between the cyst and normal gastric wall muscle layer. **(C)** Repairing gastric wall muscle layer with intermittent suture.

Gastric duplication was separated by electric hook and gauze could be used for blunt dissection to locate the boundary between the cyst and normal gastric wall muscle layer (Figures 1A,B). Then the duplication was removed completely. Maintaining a certain level of tension of the cyst is helpful to separate the cyst from the normal gastric wall tissue. Therefore, we do not routinely puncture or extract the effusion in the cyst unless there is a large GD. For cysts located on the posterior wall of the stomach, an operating forceps was routinely added in the left upper abdomen to help expose the operative field and then the cyst was resected completely. After air was injected through the gastric tube to verify that the gastric muscle layer was not perforated, 5-0 absorption sutures were used to repair the gastric muscle layer (Figure 1C).

### Literature Searching Methods

The literature on GD in children was searched from the PubMed, EMBASE, and Cochrane Library databases. The keywords that were used included GD, GD cysts, duplication, foregut, neonate, child, infant, and pediatric. The retrieval time was from 1 January 2011, to 31 December 2021. All article types (including original research, case reports, case series, etc.) were included in this study if they contained four items of the following information: (1) sex; (2) age; (3) GD type; (4) GD number; (5) GD location; (6) clinical manifestations; (7) preoperative diagnosis; (8) accompanying malformations; (9) surgical types; (10) pathological results; and (11) follow-up information. Duplications that did not occur in the stomach were excluded from this study.

### Follow-up

Follow-up examinations were performed 1, 3, 6, and 12 months after surgical completion and every 6 months thereafter. Each review included a physical examination and an abdominal ultrasound. Postoperative complications, such as bleeding, anastomotic fistula, and intestinal obstruction, were clinically evaluated in accordance with appropriate investigations.

### Statistical Analysis

Data were analyzed with the SPSS 21.0 package. The data for age and follow-up duration are expressed as medians, and the categorical variables are expressed as numbers (%). The time to oral intake and the postoperative hospital stay are expressed as the mean ± SD.

## Results

**FIGURE 2:**
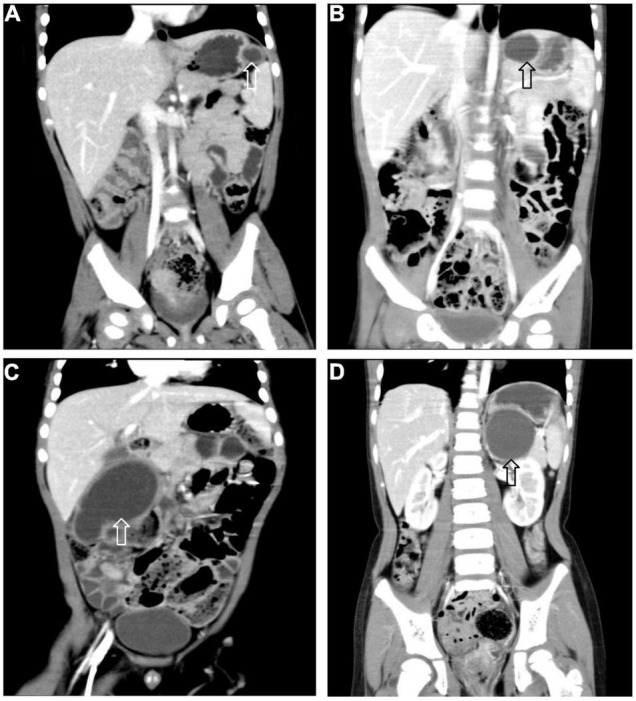
Gastric duplication (arrow) located at different sites. **(A)** Greater curvature. **(B)** Cardia. **(C)** Pylorus. **(D)** Posterior wall of stomach.

**FIGURE 3:**
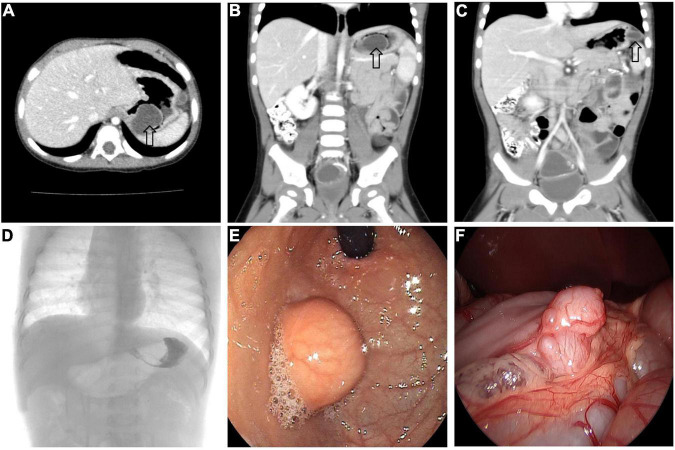
Multiple GDs (arrow) in a 1-year-old boy. **(A–C)** CT showed GDs located at the gastric funds and greater curvature. **(D)** Upper gastrointestinal radiography showed filling defect in the gastric funds. **(E)** Gastroscope found a spherical mass at the gastric fundus with a smooth surface. **(F)** An irregular mass can be seen at the great curvature of the stomach under laparoscopy.

**FIGURE 4:**
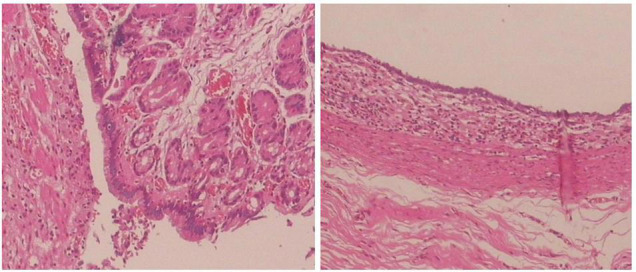
Histopathology of the resected specimen showing the cystic lesion was lined with columnar epithelial mucosa and had its own smooth muscle layer. (Hematoxylin and eosin stain, ×100).

All 17 cases of GD were cystic and had no communication with the gastric cavity. GD was located at the greater curvature of the stomach in eight cases, the cardia in two cases, the pylorus in three cases, the gastric posterior wall in three cases, and the gastric fundus in one case (Figure 2). Among them, two children had multiple GDs (Figure 3). There was one patient who had an atrial septal defect, one patient who had an indirect inguinal hernia, one patient had an accessory spleen, and one patient who had esophageal atresia. The clinical symptoms were vomiting in 2 patients (11.8%, 2/17), abdominal pain in 1 patient (5.9%, 1/17), an abdominal mass in 1 patient (5.9%, 1/17), fever in 1 patient (5.9%, 1/17), and an abdominal mass in 12 patients (70.6%, 12/17), as determined by prenatal abdominal ultrasounds. In one case of multiple GDs, part of the cyst wall on the greater curvature of the stomach was closely adhered to the splenic hilum and could not be completely removed. A mucosectomy was performed, and the remaining part of the cyst’s wall tissue that was close to the splenic hilum was electrocauterized. The GD of one of these 17 investigated cases was closely adhered to the pylorus and the boundary between the cyst and normal gastric wall muscle layer could not be identified definitely. The laparoscopy was then converted to an open operation since it was too difficult to stop bleeding during the resection. The remaining children underwent a complete laparoscopic resection of the GD. The postoperative pathology examinations confirmed GDs (Figure 4). The time for beginning oral intake after the operation was 1–4 days, with an average of 2.3 ± 1.0 days; the time of the hospital stay after the operation was 2–9 days, with an average of 5.7 ± 1.7 days. There were no complications, such as bleeding, anastomotic fistulas, gastric emptying disorders, or intestinal obstructions. After 6–77 months of follow-up, all children had an uneventful recovery with the resolution of preoperative symptoms.

By searching the PubMed, EMBASE, and Cochrane Library databases, 28 studies on GD in children were retrieved, and there was a total of 36 children. Combined with the clinical data of the 17 children treated in our hospital, we summarized the characteristics of the children’s GD (Supplementary Table 1). GD is more common in females, most of these children are diagnosed and treated before the age of 2, and the single cystic type in the greater curvature of the stomach is the most common. The most common clinical manifestation was vomiting, and the most common accompanying malformation was ectopic pancreas. The preoperative diagnosis rate of GD is low, and GD resection is most commonly used for the treatment. The follow-up outcomes showed that most of the patients were alive with no evidence of disease (ANED).

## Discussion

Gastric duplication is a rare congenital malformation of the gastrointestinal tract that accounts for approximately 2–9% of gastrointestinal malformations and is more common in females (10, 11). The etiology is not clear, but many theories have been put forward, including McLetchie’s theory, Bremer’s theory, split notochord, partial twinning theory, and intrauterine trauma (12–15). GD can be cystic or tubular in shape and can be single or multiple. Cystic lesions attached to the greater curvature of the stomach and that are not connected with the gastric cavity are the most common (16, 17). GD is often associated with other malformations, such as vertebral deformity and ectopic pancreas (18). Rare complications include lung sequestration and polycystic kidney (19). The previous literature on GD in children are mostly case reports. To our knowledge, this study is the retrospective study with the largest number of single-center clinical samples that has been described to date. Combined with the literature review, the results of this study are consistent with the previous literature.

The symptoms and signs of GD include abdominal pain, vomiting, fever, gastrointestinal bleeding, and an abdominal mass, but there is no specific symptoms. The clinical symptoms and signs of GD depend on the type, size, location, and age of onset (20, 21). Vomiting is common in children, while abdominal pain, abdominal distension, weight loss, and other symptoms are common in elderly patients. In this study, one child was treated with anti-inflammatory treatment for recurrent fever and had decreased hemoglobin, but the child did not improve. Later, CT examination results showed multiple GDs with an infection and the formation of splenic abscesses. Intraoperative findings showed that the GD on the greater curvature side of the stomach was closely adhered to the tail of the pancreas and splenic hilum, and the cyst’s wall was thickened. We considered that the fever and decreased hemoglobin in the child was due to an intracystic infection with bleeding and that bacteria had penetrated the cyst’s wall into the splenic artery, resulting in the formation of splenic abscesses. Therefore, for patients with left upper abdominal masses with fever and splenic abscesses, we should consider the possibility of a GD.

Gastric duplication has no specific clinical manifestations, and some patients may be asymptomatic. Therefore, the clinical diagnosis mainly depends on imaging examinations. (1) On ultrasound, a GD mostly manifests as a thick walled cystic mass with a clear boundary. The internal hyperechoic layer and external hypoechoic layer (double wall sign) are typical ultrasonic features (22). With the application of prenatal ultrasound, the frequency of prenatal diagnosis is also increasing. Li et al. (1) reported that the prenatal ultrasonic diagnosis rate of GD in children was 16.76%. About half of the patients in our group were diagnosed by prenatal ultrasound. Therefore, for the prenatal detection of abdominal masses, especially the left upper abdomen, we believe that ultrasound can be used as the first choice for the early screening of GD. (2) CT provides better information than ultrasound for the exact range and the edge location of the cystic mass. CT can observe the location of the GD, its relationship with the normal stomach (intragastric or extragastric) and any adhesions with surrounding tissues, which provides more information for further diagnosis and surgical planning. (3) Upper gastrointestinal radiography: radiography can evaluate filling defects or any external compressions caused by the cystic occupation. Tubular GD can be connected with the normal gastric cavity, and the contrast medium can enter the duplicate digestive tract from the normal gastrointestinal tract. This group of data could only show the filling defect in the stomach or the impression outside the gastric cavity, and there was no contrast medium flowing into the duplicate gastric cavity that was seen. This may be related to the small number of cases and the lack of tubular GD in this group of patients. (4) Endoscopic ultrasonography (EUS): EUS is a technique combining endoscopy and ultrasound, which can clearly display the digestive tract wall and its adjacent organs and tissues. The ability to distinguish solid and cystic masses is an obvious advantage of EUS. According to the source and echo characteristics of the lesion, EUS can provide preliminary information on the nature and source of the lesion. Therefore, EUS is considered to be an important tool for evaluating submucosal lesions (23). GD needs to be distinguished from other cystic masses in the upper abdomen, such as pancreatic cysts, adrenal cysts or gastrointestinal stromal tumors (24, 25). Based on the data of 17 patients and literature review, although ultrasound, CT or angiography were performed before surgery, the preoperative diagnosis rate of GD is still low and the final diagnosis of the other patients required surgical exploration. This result is similar to that reported by Li et al. (1).

Although most GDs are benign, because serious complications, such as bleeding, perforation, obstruction, and even malignant transformation need to be considered, most studies recommend surgical resection once the diagnosis is made. For cystic GD, surgical treatment should be selected according to the GD type, but tubular GD usually does not require any intervention (26). The surgical treatment includes laparoscopic cyst resection, open cyst resection, partial gastrectomy, mucosal dissection, and endoscopic resection (5, 27). Fang et al. (28) reported that endoscopic ultrasound submucosal resection of an infant GD has a good effect. In this study, one child’s GD was located at the gastric fundus, but we did not use endoscopic submucosal dissection (ESD), as this modality was not available at our institution. Considering that if only marsupialization is performed, residual food may still be present after the operation, resulting in diverticulitis, so we performed a laparoscopic GD resection. Then, the child recovered well after the operation. In this study, all children, except for one, had their GD completely removed under laparoscopy, and the child had multiple GDs, which could not be completely removed due to the close adhesion between part of the cyst’s wall on the side of the great curvature and the splenic hilum, which caused a small amount of cyst wall to remain. During the operation, we had three experiences as follows: (1) In case of exposure difficulties during the operation, 3-0 Prolene sutures could be used to suspend the gastric wall, or operating forceps could be used in the left upper abdomen to help expose the operative field. (2) If there were no complications such as infection, bleeding or perforation happened, the boundary between the duplicate cyst and normal stomach wall muscle layer can be found by blunt and sharp separation alternately, and the GD can be completely removed. (3) It is generally not recommended to puncture or extract the effusion in the cyst unless there is a large GD. Maintaining a certain level of tension of the cyst is helpful for the surgeon to separate the cyst from the normal gastric wall tissue. If the cyst wall is thin and breaks during the separation, the cyst wall can be opened, and gauze can be placed into the cyst cavity to play a supporting role to facilitate the identification of the tissue gap between the GD and the normal gastric wall muscle layer.

The limitations of this study were: (1) Although this study is a retrospective study with the largest number of samples reported by a single center, the sample size is still small, and the literature review time is limited. Therefore, the results still need to be interpreted with caution. (2) The long-term efficacy needs to be evaluated in children using long-term follow-up.

## Conclusion

Gastric duplication in children is rare and has no specific clinical manifestations. The diagnosis requires surgical exploration combined with a pathological examination. This disease can lead to serious complications, so surgery should be performed as soon as possible. Laparoscopic resection of GD can achieve good therapeutic results.

## Data Availability Statement

The original contributions presented in the study are included in the article/Supplementary Material, further inquiries can be directed to the corresponding author.

## Ethics Statement

The studies involving human participants were reviewed and approved by the Institutional Review Board of Guangzhou Women and Children’s Medical Center. Written informed consent to participate in this study was provided by the participants’ legal guardian/next of kin.

## Author Contributions

FL and JZ contributed to the study conception and design. XX, ML, BT, and ZL performed the material preparation, and data collection and analysis. FL wrote the first draft of the manuscript. All authors commented on previous versions of the manuscript, read and approved the final manuscript.

## Conflict of Interest

The authors declare that the research was conducted in the absence of any commercial or financial relationships that could be construed as a potential conflict of interest.

## Publisher’s Note

All claims expressed in this article are solely those of the authors and do not necessarily represent those of their affiliated organizations, or those of the publisher, the editors and the reviewers. Any product that may be evaluated in this article, or claim that may be made by its manufacturer, is not guaranteed or endorsed by the publisher.
