# Supplementary material for: Clinical Characteristics of Gastric Duplication in Children
Source: Front Pediatr. 2022 Mar 28;10:857056. doi: 10.3389/fped.2022.857056 (PMC8995966; doi:10.3389/fped.2022.857056)
Supplement: Supplementary file 1 [file Table_1.DOCX]

Supplementary Table 1. Clinical characteristics of gastric duplication in children

|  | Literature review patients(n=36) | Our group patients(n=17) | All included patients(n=53) |
| --- | --- | --- | --- |
| **Sex** | Valid=27 | Valid =17 | Valid =44 |
| Female | 17(62.96%) | 9（9/17） | 26（**59.09%**） |
| **Age（months）** | Valid=33 | Valid=17 | Valid=50 |
| Range | 0-120 | 2-132 | 0-132 |
| Median | 12 | 24 | **13.5** |
| **Type** | Valid=31 | Valid=17 | Valid=48 |
| Cystic | 28（90.32%） | 17（17/17） | 45（**93.75%**） |
| Tubular | 3（9.68%） | 0（0/17） | 3（6.25%） |
| **Number** | Valid=36 | Valid=17 | Valid=53 |
| Single | 34（94.44%） | 15（15/17） | 49（**92.45%**） |
| Multiple | 2（5.56%） | 2（2/17） | 4（7.55） |
| **Sites** | Valid=28 | Valid=17 | Valid=45 |
| Greater curvature | 11（39.29%） | 8（8/17） | 19（**42.22%**） |
| Lesser curvature | 1（3.57%） | 0（0/17） | 1（2.22%） |
| Funds | 2（7.14%） | 1（1/17） | 3（6.67%） |
| Cardia | 3（10.71%） | 2（2/17） | 5（11.11%） |
| Pylorus | 11（39.29%） | 3（3/17） | 14（31.11%） |
| PW | 0（0%） | 3（3/17） | 3（6.67%） |
| **Clinical manifestations** | Valid=34 | Valid=17 | Valid=51 |
| **Symptomatic** | 29（85.29%） | 5（5/17） | 34（66.67%） |
| Vomiting | 17（58.62%） | 1（1/5） | 18（**52.94%**） |
| Abdominal pain | 6（20.69%） | 2（2/5） | 8（23.53%） |
| Gastrointestinal bleeding | 5（17.24%） | 1（1/5） | 6（17.65%） |
| Abdominal mass | 1（3.45%） | 0（0/5） | 1（2.94%） |
| Fever | 0 | 1（1/5） | 1（2.94%） |
| **Asymtomatic** | 5（14.71%） | 12（12/17） | 17（33.33%） |
| **GD diagnosis-Preoperative** | Valid=34 | Valid=17 | Valid=51 |
| Yes | 14（41.18%） | 8（8/17） | 22（**43.14%**） |
| No | 20（58.82%） | 9（9/17） | 29（56.86%） |
| **Accompanying malformations** | Valid=20 | Valid=4 | Valid=24 |
| Ectopic pancreas | 9（45%） | 0（0/17） | 9（**37.5%**） |
| Other sites duplications | 2（10%） | 0（0/17） | 2（8.33%） |
| Congenital heart disease | 5（25%） | 1（1/17） | 6（25%） |
| Vertebral abnormalities | 1（5%） | 0（0/17） | 1（4.17%） |
| Other anomalies | 3（15%） | 3（3/17） | 6（25%） |
| **Surgical types** | Valid=36 | Valid=17 | Valid=53 |
| **Open surgery** | 24（66.67%） | 1（1/17） | 25（47.17%） |
| Cystectomy | 20（83.33%） | 1（1/1） | 21（84%） |
| Mucosectomy | 1（4.17%） | 0（0/1） | 1（4%） |
| Cystectomy+Mucosectomy | 1（4.17%） | 0（0/1） | 1（4%） |
| Partial gastrectomy | 2（8.33%） | 0（0/1） | 2（8%） |
| **MIS** | 12（33.33%） | 16（16/17） | 28（52.83%） |
| LAP cystectomy | 8（66.67%） | 15（15/16） | 23（82.14%） |
| LAP cystectomy+gastrostomy | 1（8.33%） | 0（0/16） | 1（3.57%） |
| LAP cystectomy+pyloroplasty | 1（8.33%） | 0（0/16） | 1（3.57%） |
| LECS | 1（8.33%） | 0（0/16） | 1（3.57%） |
| LAP cystectomy+Mucosectomy | 0（0%） | 1（1/16） | 1（3.57%） |
| ESD | 1（8.33%） | 0（0/16） | 1（3.57%） |
| **Follow-up** |  |  |  |
| **Duration（months）** | Valid=13 | Valid=17 | Valid=30 |
| Range | 0.25-168 | 6-77 | 0.25-168 |
| Median | 6 | 17 | 14 |
| **Outcomes** | Valid=32 | Valid=17 | Valid=49 |
| ANED | 31（96.88%） | 17（17/17） | 48（**97.96%**） |
| DOD | 1（3.12%） | 0（0/17） | 1（2.04%） |

PW, posterior wall of stomach; MIS, minimally invasive surgery; LAP, laparoscopic; LECS, laparoscopic-endoscopic cooperative surgery; ESD, endoscopic submucosal dissection; ANED, alive with no evidence of disease; DOD, dead of disease.
